# Volcanic ash in the air we breathe

**DOI:** 10.1186/2049-6958-8-52

**Published:** 2013-08-07

**Authors:** Bernadette M Longo, Anthony A Longo

**Affiliations:** 1 Orvis School of Nursing, University of Nevada, Reno 0136, NV, USA; 2 Department of Geosciences, University of Nevada, Las Vegas, NV, USA

## 

Earth is no stranger to volcanism. Since proto-Earth and crustal formation, volcanoes have provided pathways for degassing and cooling of our planet’s interior. Volcanism played an important role in the formation of the early atmosphere, oceans and continents, and was likely a necessary component for the development of life. Throughout our species brief history, innumerable volcanoes have erupted, and many are the same volcanoes still active today. Volcanic eruptions have triggered the demise of ancient civilizations, and persist as an immense force that dwarfs modern technology. They are uncontrollable, unpredictable, and catastrophic to populations and modern cities built along their flanks. Eruptions cause adverse effects ranging from disruption of air travel, disturbance in local ecosystems, to profound changes in global climate. On average, 50 volcanoes erupt each year [1], thus reminding us that our planet is alive and constantly changing. Globally, nearly 1 in 10 persons live within 100 km of a historically active volcano [2]. Communities that live through a volcanic eruption can experience havoc, fear, loss of home and livelihood and suffer increased morbidity and mortality.

**Figure 1:**
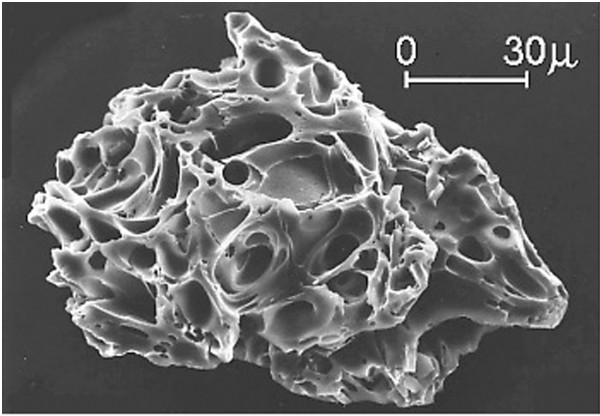
**SEM image of volcanic ash from Mount St. Helen’s eruption.** Credit: United States Geological Survey and A.M. Sarna-Wojcicki.

Ash fall is a hazardous product of volcanic eruption that can affect large populations and create widespread economic chaos. Volcanic ash particles are fine-sized (<2 mm), pyroclastic fragments that form during eruption as gases exsolve from the magma, develop large bubbles that burst causing fragmentation, and are explosively propelled upward. Subsequent explosions violently shatter the surrounding vent rock and hurl it as fragments into the air. Once airborne, plumes of ash are subject to meteorological effects such as dispersion with wind and removal by rain. The severity of an eruption is a function of the volcanic explosivity index (VEI, range 0 to 8) [3]. Eruptions with low VEI’s 0–2 build volcanoes that passively de-gas and erupt lava effusively; whereas, high VEI’s are characteristic of explosive volcanism. The higher the VEI the greater the volume of ejecta, the greater degree of fragmentation creating more toxic fine ash, the higher the eruption column, and the greater distances large volumes of ash can travel. After eruption, deposits of ash are remobilized by wind or human activities for decades. Hence, the health risk from exposure is not limited to the timeframe of eruption, but may continue long after volcanic activity has ceased.

An analysis of the characteristics of volcanic ash conducted by volcanologists is necessary to assess the health hazard on exposed populations. The small particle size allows penetration into the alveolar region of the lung. The reactivity of ash particles with lung tissue depends on the morphology, surface area and number of particles [4]. Toxicity of the ash is dependent on the percent concentration of the crystalline silica polymorphs present in the ash column. Quartz is most abundant, but tridymite and cristobilite are most toxic and damaging to the lung tissue. The composition of the eruptive ejecta with regards to the weight percent (wt%) SiO_2_ has implications towards the concentration of crystalline silica in the eruption column. Explosive eruptions with compositions >60 wt% SiO_2_ have higher concentrations of crystalline silica, whereas basaltic (40-50 wt% SiO_2_) eruptions lack crystalline silica. Moreover, unweathered crystalline silica readily develops jagged shards of glass that will remain lodged in, and therefore, toxic to lung tissue. Exposure to these toxic polymorphs may instigate a pathological cycle in the lung; persistent inflammation, macrophage death, and abnormal production of collagen resulting in development of fibrotic tissue [4]. New data indicate that toxicity through surface reactivity with iron-rich basaltic ash or minerals is enhanced through hydrolysis and oxidation in the lungs creating free radicals and toxic reactions as dangerous as the crystalline silica [4]. In addition, acidic gases emitted during an eruption can adsorb onto unaltered ash particles, further contributing to the toxicity and pathology caused from exposure.

Since the turn of the century, the causal links between exposure to fine-sized particles of urban pollution and cardiorespiratory effects are better understood. Inhalation of fine-sized particles can affect health by increasing oxidative stress, systemic inflammation, endothelial dysfunction, blood viscosity, pro-atherothrombosis, blood pressure and autonomic dysfunction of the heart. Increased cardiovascular mortality is associated with exposure to fine-sized particles, noting most deaths are from ischemic events [5]. Volcanic-derived fine particles are different in morphology and chemistry from anthropogenic particles. From a health perspective, there are likely similarities in the disease burden on exposed populations. Undoubtedly, more multidisciplinary studies are needed in the emerging area of volcanic health research.

A new study at Mount Etna by Lombardo et al*.*[6] published in this issue of *Multidisciplinary Respiratory Medicine* contributes to the understanding of adverse health effects caused by exposure to volcanic ash. Etna is a large stratovolcano in the Catania Province, Italy, with one of the longest documented records of eruption. Two styles of eruptive activity typically occur (VEI 1–3); persistent explosive eruptions from one or more of the four summit craters, or less frequent effusive eruptions with lava fountaining along the flanks of the volcano [7]. Both summit and flank eruptions have produced ash, and thereby pose potential health risks to nearby residents. Already this year, there have been 13 episodes of lava fountaining from Etna (as of July 2013). These researchers re-evaluated the burden of cardiorespiratory presentations to local hospitals during the historical 2002 eruption. This new work builds on knowledge gleaned from a previous study [8] and provides new insight into the public health burden as well as evidence-based recommendations for future eruptions. The study [6] identifies an increase in patients with acute cardiorespiratory illnesses presenting to emergency departments during ash fall. Most interesting were the significant cardiovascular, upper and lower respiratory and ocular effects relative to geographic exposure. These findings are biologically plausible and consistent with the previously identified [8] increase in emergent presentations for ischemic heart and myocardial infarction. Lombardo et al. [6] offer a perspective on implications for occupational health and recommend future health prevention efforts for the highly populated province.

An increased burden of acute illnesses should be anticipated by health care agencies in communities impacted by volcanic ash fallout. Health care clinicians practicing in areas near active volcanoes in Italy and around the world have an opportunity to work together with multiple disciplines to increase the public’s awareness of volcanic hazards, provide interventions across levels of prevention, and advocate to authorities on behalf of their patients. The multi-disciplined “International Volcanic Health Hazard Network” offers resources for professionals and the public [9]. Further studies are needed on acute and residual effects of major eruptions that can initiate public health interventions, disaster planning and multi-disciplinary efforts to enhance population health in volcanic areas around the world.
